# The Late Sir Felix Semon

**Published:** 1921-04-02

**Authors:** 


### The Late Sir Felix Semon.

A REMINISCENCE.

The occasion was a meeting of the Laryngological Society—then newly transformed into the laryngological section of the Royal Society of Medicine—and the place, of course, London. The period was about 1908. A big room with rather dim throat-lamps dotted round it; biggish table in the middle with microscopes; platform on the left as you entered, for the chairman. There were present nearly all the prominent members of the specialty, engaged, before the meeting began, in looking down the microscopes aforesaid, and also the throats, noses, and ears of "cases," by aid of the aforesaid lamps. Sir Felix troubled himself only with the throats and took an exceedingly rapid survey. He was a man of medium height and rather strong features, alert-looking, more of the man of action than of the savant. Dark complexioned, his hair had turned grey, but not his eyebrows and pronounced moustache; he was obviously a foreigner before he spoke. And this he did only when laryngeal affections were discussed; but then first of all and with an intonation of authority. There was a patient with suspected cancer, shown by a well-known laryngologist, who said that the epiglottis overhung a little, making inspection rather difficult. Semon had noticed no difficulty at all; he had also examined under the microscope the section of an excised piece. These appearances he pronounced suspicious, but "not conclusive." With these two last words, delivered with emphasis and finality, unfairly exaggerated by his strong foreign voice, he sat down. Whereupon there got up one of pertinacious and radical aspect, who presently took occasion to remark, lowering his tone noticeably at the contradiction, and assuming a sly and subacid expression, that he also had seen the section, but to him the appearances *were* conclusive. One divined somehow that he knew Sir Felix was getting a little deaf. If so, he was successful in "drawing" the celebrity, who exclaimed strongly "Speak up!" but without producing the smallest effect upon the speaker. The next participator in the discussion was a young North Briton. With national caution he expressed a slight dissent from the famous man, but added, with an engaging smile and a little ostentation—as seeking to allay the tiny breach of harmony that had just occurred—that he should not dream of disputing with an authority like, etc., etc. The room had not long to wait to see who was right and who was wrong. Someone asked for opinions on a larynx with a queer tumour, lying on a plate. Everybody had looked at it; but nobody seemed to want to risk putting a name to it; so there was silence. Suddenly Sir Felix said, "What nationality was the patient?" Glances slightly apprehensive, as of impending eye-wiping, were exchanged. The reply "From Ceylon" caused this apprehension to begin to pass into resignation and acceptance of defeat, a change quickly completed by the triumphant rejoinder, "It is right; they have it there: it is scler-r-ôma" (the Central European, and also by the way Northumbrian, uvular R well in evidence). There were no further comments on this case, and none seemed needed. The old master was still the master; and his trifle of over-eagerness to demonstrate this, his carelessness of oto-rhinology, his foreign blood, combined to give a quality of dramatic antagonism to that session which one observer, at any rate, is glad not to have missed. It was but a few years later that he retired.

